# Application of multiplex PCR for Rapid and sensitive detection of human papillomaviruses in cervical cancer

**DOI:** 10.12669/pjms.322.8582

**Published:** 2016

**Authors:** Fateme Zandnia, Abbas Doosti, Abbas Mokhtari-Farsani, Mohammad Taghi Kardi, Abolfazl Movafagh

**Affiliations:** 1 Fateme Zandnia, Biotechnology Research Center, Islamic Azad University, Shahrekord Branch, Shahrekord, Iran; 2 Abbas Doosti, Biotechnology Research Center, Islamic Azad University, Shahrekord Branch, Shahrekord, Iran; 3 Abbas Mokhtari-Farsani, Young Researchers and Elite Club, Biotechnology Research Center, Islamic Azad University, Shahrekord Branch, Shahrekord, Iran; 4 Mohammad Taghi Kardi, Department of Biology, University of Isfahan, Isfahan, Iran; 5 Abolfazl Movafagh, Department of Medical Genetics, Pediatric Neurology Research Center, School of Medicine, Shahid Beheshti University of Medical Sciences, Tehran, Iran

**Keywords:** Human papillomavirus, Cervical cancer, Multiplex PCR, Biomarker

## Abstract

**Objectives:**

Reffering to an increase in cervical cancer in the recent years, rapid, sensitive and economical detection of human papillomaviruses (HPVs) as causative agents of cervical cancer is important. The traditional methods for the detection of HPVs in cervical cancer, such as pap smear, suffer from limitation and PCR has a potential to overcome the limitaitons. The purpose of present research work was to identify the five important strains of HPV (16, 18, 31, 33 and 45) simultaneously by Multiplex PCR application.

**Methods:**

Study was done on 100 cervical lesions of women. DNA was extracted from specimens by a genomic DNA purification kit. A 5-plex PCR was developed for the simultaneous detection of major HPV. Five pair of new primers was designed for detection of HPV 16, 18, 31, 33 and 45 by Multiplex PCR.

**Results:**

Among the 100 evaluated samples, 82 were found positive to HPVs. In the meantime the highest rate of infection was for HPV 16. Also 30 of HPV positive samples had infections with two or more HPV types.

**Conclusion:**

Multiplex PCR assay used in present study can provide a rapid, sensitive and economical method for detection of viral infections and is applicable to small volumes of vaginal samples.

## INTRODUCTION

Human Papilloma Viruses (HPVs) are agents causing cervical cancer that have been detected in up to 99.7% of cervical squamous cell carcinoma and 94 to 100% of cervical adenocarcinomas.^1^ Persistent infection by certain genotypes of HPV plays a crucial role in createing tumors.^2^ HPVs are members of the papovaviridae,^3^ consist of almost 8000 bp long circular DNA molecular that are wrapped into a protein shell which is composed of two molecules (L1 and L2). In addition, the genome has the coding capacity for six proteins, so-called early proteins (E1, E2, E4, E5, E6 and E7). These proteins are necessary for viral replication and for assembly of newly produced virus particles in the infected cells. There is an upstream regulatory region (URR) of 1000 bp without coding any proteins, that separates early and late genes. URR contains cis-elements required for regulation of gene expression, replication of the genome and packaging into virus elements. Over 200 types of HPV have been known and about 40 types are able to infect the genital organ.^4^ HPVs can be classified into high or low risk types depending upon their oncogenic potential. High-risk genotypes consist of 16, 18, 31, 33, 35, 39, 45, 51, 52, 53, 56, 58, 59, 66, 68, 70, and 73 that 16 and 18 are associated with 70% of cervical cancer.^5^ Low risk HPV such as HPV6 and HPV 11 were first cloned from genital warts or condylomata acuminate.^6^ Investigations have shown that a range from 2% to 20% of women have detectable HPV-DNA in their cervix at any time.^7^

Cervical cancer is the second most common cancer that affects more than four hundred thousand women each year and a leading cause of cancer-related death worldwide.^8^ Although the incidence and mortality from cervical cancer have markedly decreased due to high-quality screening with cytology and the development of cervical vaccines, but the disease burden remains significant with 530,000 new cases and 275,000 deaths from cervical cancer in 2008.^9^ Currently known co-factors associated with cervical cancer development are cigarette smoking, alcohol consumption, micro nutrients deficiency in fruits and vegetables, prolonged use of oral contraception, multiparity, uncircumcised male partner, low socioeconomic status (SES), infection with HIV/AIDS or other STIs including herpes simplex and chlamydia trachomatis.^10^

HPV cannot grow in conventional cell culture, and serological assays have only limited accuracy. Cervical cancer screening is currently with the papnicolaou (pap) smear which has had a significant impact on the reduction in the incidence of cervical cancer. However, the sensitivity of cytological test is variable greatly according to experience of the cytologist and other conditions.^8^ Since Polymerase chain reaction (PCR) has the potential to overcome pap smear limitation, and Multiplex PCR is a variant of PCR that several target sequences are simultaneously amplified in the same reaction, therefore, the purpose of the present study was increasing the sensitivity and shortening the time of diagnosis of HPV infection by Multiplex PCR assay.

## METHODS

### Clinical specimens

Those women who had cervical lesion in refferal and teaching hospital affiliated to Isfahan University of Medical Sciences (in central of Iran) were included in the study. One hudnred samples were collected (during winter season 2014). All samples were taken using of sterile cotton swab, and were stored at -20ºC until required for DNA extraction. Permision from the ethical committee was taken. The anonymity of participants and data confidentiality was protected. Protocols and approved proposal according to local law and regulations, by the Institutional Review Boards of each participating referral hospital.

### Primers Design

The gene bank accession for the DNA sequences used, are shown in Table-I. Highly conserve regions with E6 HPV viruses (16, 18, 31, 33 and 45) were determined. All primers were designed from regions with least homology to other HPV viruses. Primers sequences that were used in present study are shown in Table-I. The primers were design using Gene Runner software (Version 3.01) and all sequences were checked for hairpin loop, dimer formation and convenient and near annealing temperature. The accuracy of designed primers was confirmed by Blast program (http://wwwncbi.nlm.nih.gov/BLAST/).

**Table-I:** Primers sequences for detection of HPVs.

Primer name		Oligonucleotide sequence (5’ to 3’)	Size	Accession Number
HPV 16 (E6)	F	5’- TTGCTTTTCGGGATTTATGC -3’	202 bp	FJ610152
	R	5’- GGACACAGTGGCTTTTGACAG -3’		
HPV 18 (E6)	F	5’- CAGAAACCGTTGAATCCAGCAG -3’	279 bp	KC470213
	R	5’- CATCGTTTTCTTCCTCTGAGTCG -3’		
HPV 31 (E6)	F	5’- AAGGTCAGTTAACAGAAACAGAGG -3’	339 bp	J04353
	R	5’- TTTCAGTACGAGGTCTTCTCCAAC -3’		
HPV 33 (E6)	F	5’- CAACATTGAACTACAGTGCGTGG -3’	154 bp	M12732
	R	5’- TTCACTAATTTTAGATAAGAACCGC -3’		
HPV 45 (E6)	F	5’- ATGTGTAGGTATGGAAATTGGTCG -3’	451 bp	KC470260
	R	5’- CAAACAGTTGTTCACGGCGTAG -3’		

### DNA extraction protocol

The sterile cotton swabs were placed in 1.5 ml sterile tubes and viral DNA was extracted by a genomic DNA purification kit (CinnaGen Co, Iran) according to the manufacturer’s recommendation. The extracted DNA was quantified by spectrophotometric measurement at a wavelength of 260 nm and was kept frozen at -20°C until used.

### Multiplex PCR amplification conditions

A 5-plex PCR was performed for identification of HPV-16, 18, 31, 33 and 45. The Multiplex PCR was run in final reaction volumes of 50 μl containing the following reagents: 100 ng of genomic DNA, 50 mM KCl, 10 mM Tris-HCl (pH 8.5), 6 mM MgCl2, 200 μM each of dATP, dCTP, dGTP and dTTP, 50 pmol of each primer and 5 unit of Taq DNA polymerase (CinnaGen Co, Iran). The PCR assay was performed at 95ºC for 6 minutes (pre denaturation) and then for 40 cycles of 94ºC for one minutes, 60ºC for one minutes, 72ºC for minutes, with final extension at 72ºC for seven minutes in a thermal cycler (Mastercycler gradient, Eppendrof, Germany), Negative controls were included in each run contain HPV 54.

### Detection of PCR products

The PCR-amplified products were detected in 1.5% ethidium bromide (EtBr)-stained agarose gel electrophoresis. Aliquots of 15 μL of PCR products were applied to the gel. Voltage of 120 V for two minutes and then 85 V for 25 minutes was used for products separation. The DNA molecular weight marker (100 bp, Fermentas, Germany) was used as a size marker. After electrophoresis, images were obtained in UVIdoc gel documentation systems (UK).

### Statistical Analysis

For presence of HPVs (16, 18, 31, 33 and 45) all data were analyzed by MS Excel 2007 and the Chi-square test using the SPSS 17 (SPSS Inc. Chicago, IL, USA) software. *P* values <0.05 were considered significant.

## RESULTS

**Fig.1:**
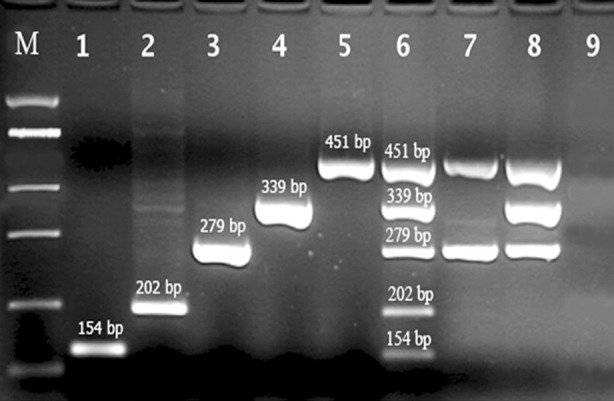
Agarose gel electrophoresis of 5-plex PCR amplification products for detection of HPV-types (Lane M shows fermentas 100 bp DNA molecular marker, Lane 1 is positive sample for HPV 33, Lane 2 is positive sample for HPV 16, Lane 3 is positive sample for HPV 18, Lane 4 is positive sample for HPV 31, Lane 5 is positive sample for HPV 45, Lane 6 is positive sample for all five types of HPV, Lane 7 is positive sample for HPV 18 and 45, Lane 8 is positive sample for HPV 18, 31 and 45, and Lane 9 is negative control contain HPV 54)

One hundred samples of mucosal cervical lesion from women were examined for presence of HPVs DNA (16, 18, 31, 33 and 45). The primers used were derived from the E6 HPV gene of HPVs. Agarose gel electrophoresis of positive samples revealed 202 bp fragment (HPV 16), 279 bp fragment (HPV 18), 339 bp fragment (HPV 31), 154 bp fragment (HPV 33) and 451 bp fragment (HPV 45). An example of Multiplex PCR amplification of mucosal samples is shown in Fig.1. HPVs DNA were found in 82 of 100 (82%) mucosal samples, including 60 HPV16, 28 HPV 18, 4 HPV 31, 2 HPV 33 and 4 HPV 45. Also 30 (36.58%) of HPV positive samples had infections with two or more HPV types.

## DISCUSsION

An increasing number of malignancies are directly or indirectly the result of viral infection. Cervical cancer is a worldwide public health problem among women, especially in emerging nations.^11^ HPV is currently one of the most common sexually transmitted infections worldwide. Most studies in the world have demonstrated that detection of HPV DNAs in cervical samples has significantly contributed to increasing the sensitivity of the screening for cervical abnormalities, understanding the natural spread of the viral infection, and identifying carcinogenic HPV types.^12^ Typing of amplified fragments is usually performed by different assays such as, direct DNA sequencing,^13^, ^14^ restriction fragment length polymorphisms (RFLP),^15^ and hybridization to specific probes.^16^ PCR with consensus primers can potentially detect most mucosal HPV types.^17^ In present study we describe a 5-plex PCR for the specific diagnosis of five types of most important HPVs in cervical cancer. Using this method, we could identify five types of most important HPVs (16, 18, 31, 33 and 45) in cervical cancer by using five specific pair primers in one reaction, as a result, the sensitivity was increased and also the time of detection was short. Some studies were performed about comparison of methods in detection of HPV-types infection. In a study by Maranga et al., 2013 the sensitivity of Multiplex PCR and pap smear in detection of HPV type. The result of this study showed more sensitivity of Multiplex PCR as compared with pap smear.^18^ In Snijders et al., 2010 research study diagnosed the HPV DNA and RNA using southern blot and northern blot, that although the method was successful but because of the high volume of material and high costs they declared that the Multiplex PCR is better than these two method.^19^ Yet another study showed the accuracy was higher than 80% in identifying oropharynx cancer by Multiplex PCR.^20^, ^21^ Our study and other studies suggests that a Multiplex PCR assay with suitable specific primers used in present study can potentially have an impact in HPV screening.

## CONCLUSION

This study describes a rapid and sensitive assay for detection of mucosal high-risk HPV types with a high specificity for the detection of several mucosal high-risk HPV types in women at increased risk of cancer and also infected with single and multiple HPV types. We suggest use of this assay for epidemiological studies that aim to determine the distributions of various HPV types in different parts of the world.

## List of abbreviations

Human papillomaviruses (HPVs),

Upstream regulatory region (URR),

Papnicolaou smear (Pap smear).
